# Supplementary material for: Machine learning for buildings’ characterization and power-law recovery of urban metrics
Source: PLoS One. 2021 Jan 28;16(1):e0246096. doi: 10.1371/journal.pone.0246096 (PMC7842992; doi:10.1371/journal.pone.0246096)
Supplement: Attachment — Submitted filename: Reply2.pdf [file pone.0246096.s002.pdf]

We thank the Editor for seeing merit in our work and for sending us the Referees' reports. We also thank the Referees positive comments on the revised version of the manuscript, which is seen to have "largely been improved in my opinion." quoting the first Reviewer, "All comments have been addressed" as the second Reviewer states.
Concerning the detailed comments, we address them below and list the corresponding changes in the manuscript. Original comments of the first Referee are in blue and changes in the manuscript are in red, both here and in the revised manuscript.

With these changes and clarification, we trust our manuscript is now suitable for publication in PLOS One.

Reply to the first Referee:

Referee 1: The diagonal of the scatter plot matrix could be used to show the distribution of the two classes. It would add valuable information to the figure. An example of such a plot can be found at, for example, $https://seaborn.pydata.org/examples/scatterplot_{ma}$

Reply: We agree with the Referee and we thank him/her for the reference he/she provided us with. We added the below scatter plot matrix.

Action: The below figure was added along with the sentence "To visualize the outliers, the six features were reduced to three using the Principle Component Analysis (PCA). The correlations between each new dimension and the two others were then illustrated using a two-dimensional scatter plot matrix shown in Fig. 1. The diagonal plots show the univariate distribution of each dimension."

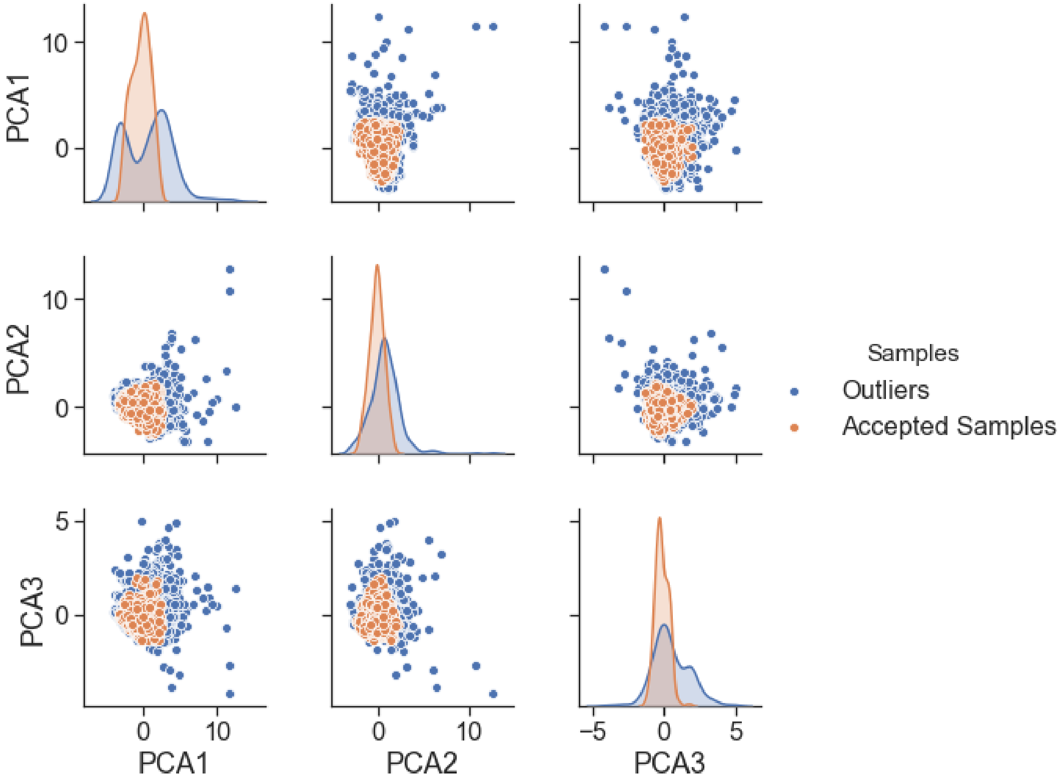

Correlation plot of the buildings samples after applying PCA for dimension reduction, with outliers highlighted in blue.

Referee 1: It would be, additionally, be very informative to have a map similar to figures 3 and 4 with the buildings that are actually used in the analysis.

Reply: Indeed we agree that the suggested map would inform the reader about the used buildings in the analysis.

Action: The below figure was added along with the sentence "To visualize the outliers, the six features were reduced to three using the Principle Component Analysis (PCA). The correlations between each new dimension and the two others were then

illustrated using a two-dimensional scatter plot matrix shown in Fig. 1. The diagonal plots show the univariate distribution of each dimension. The spatial distribution of the buildings used in the development of the predictive algorithms is shown in Fig. 2."

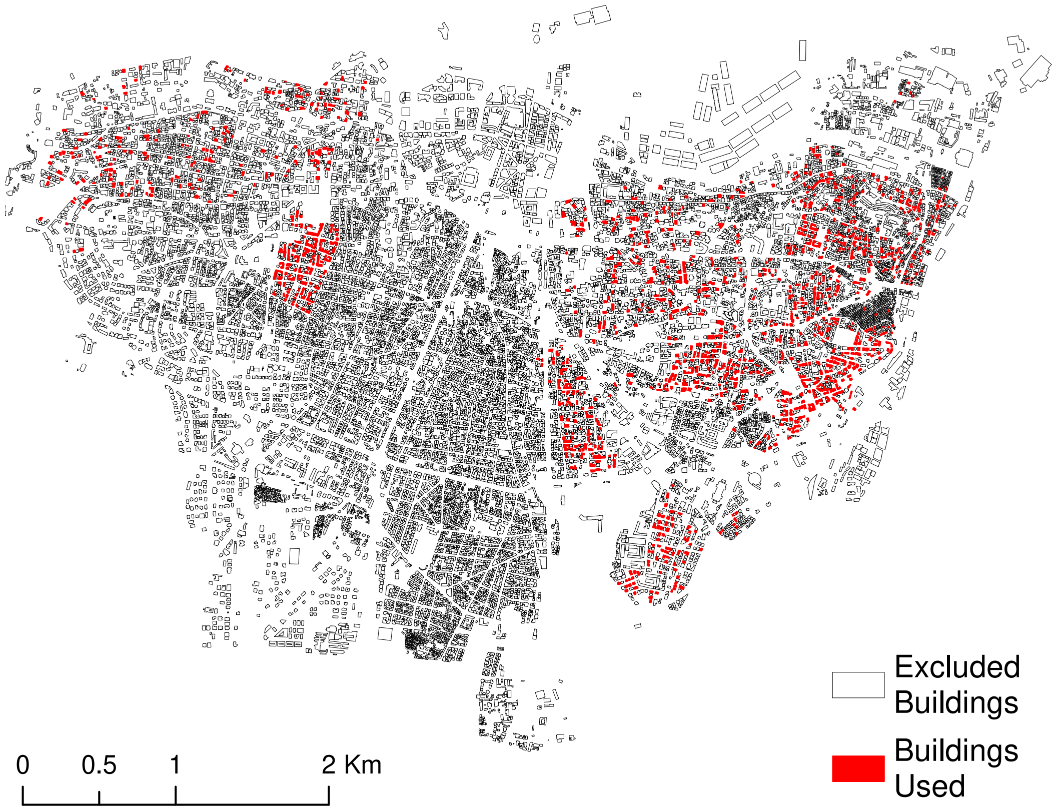

Spatial distribution of the accepted buildings after the data pre-processing.

Referee 1: Since all information is contained in figure 2, I would recommend removing figure 1, as it does not provide any useful additional information. But this I leave to the authors.

Reply/Action:
We agree with the Referee that the information showed in the two figures is redundant, especially after updating the scatter plot. The figure illustrating the 3D correlations between PCA dimensions was removed.

Referee 1: Please provide a few references to the added part in lines 136-137 to justify the percentages used.

Reply: We thank the Referee for highlighting the need for references. The paper of A. Clark entitled "The machine learning audit- CRISP-DM Framework" was added.

Action: The reference was added along with the sentence "The dataset of $1,536$ samples was subdivided into training, validation, and test sets each containing respectively 859, 369, and 308 samples, which correspond respectively to the 55%, 25%, and 20% splits, often recommended in the literature [31]."

Referee 1: In line 202 you mention that you recover known properties about heights. Please add a sentence what these properties are or add references.

Reply/Action: We thank the Referee for asking for clarification. Indeed the sentence is not clear.

Action: We added the following to the text: The fact that the distribution of predicted buildings' heights follows a power-law and not a log-normal is a confirmation that our model recovers known properties about the heights; namely that they follow a

power-law and not a log-normal distribution.

Referee 1: I apologize reiterating this point again, but in the case of buildings, what does it mean that the underlying processes are either multiplicative or additive? I personally have no intuition what that means in terms of building heights. Please clarify further.

Reply: We thank the Referee for his/her care for clarity. In terms of distribution, we a power-laws mathematically arise when the underlying process is multiplicative. This reference includes the mathematical details of our statement. Mitzenmacher, Michael. "A brief history of generative models for power law and lognormal distributions." Internet mathematics 1.2 (2004): 226-251.

Action: Rereading the statement we made about the multiplicative processes we see that it has no relevance to the flow of idea and thus decided to remove it.

Referee 1: I appreciate that you added the section from line 100 and onward. However, it does still not clarify why MLF-NNs are a good choice for the analysis you did. I'm no expert in machine learning, so please make this point a little clearer for people like me.

Reply: Many machine learning algorithms are available with different architectures. In our manuscript, we chose three well-known algorithms (linear regression, NN, and RF). Each model with a given architecture learns its parameters based on the training set. After that, to evaluate the model's performance, and thus to choose the best among them, a performance metric is applied to compare how close the actual data is to the model's prediction. This metric is normally a measure of error, or how far the predictions are from the actual data and thus the algorithm with the best metric value is chosen. After running the algorithms with our dataset, the NN algorithm described in the manuscript performed better than all the other. Therefore, it was considered the best choice for our analysis.

Action: The following sentence was added at the beginning of the Methods section: Many machine learning algorithms are available with different architectures. In our manuscript, we chose three well-known algorithms (linear and logistic regression, NN, and RF) that are described in more details below. Each model with a given architecture learns its parameters based on the training set. After that, to evaluate the model's performance, and thus to choose the best among them, a performance metric is applied to compare how close the actual data is to the model's prediction. This metric is normally a measure of error, or how far the predictions are from the actual data and thus the algorithm with the best metric value is chosen.
